# Predictors for independent external validation of cardiovascular risk clinical prediction rules: Cox proportional hazards regression analyses

**DOI:** 10.1186/s41512-018-0025-6

**Published:** 2018-02-06

**Authors:** Jong-Wook Ban, Richard Stevens, Rafael Perera

**Affiliations:** 1 Evidence-Based Health Care Programme, Centre for Evidence-Based Medicine, University of Oxford, Oxford, UK; 2 Nuffield Department of Primary Care Health Sciences, Medical Science Division, University of Oxford, Oxford, UK

**Keywords:** Clinical prediction rule, Cardiovascular disease risk, Independent external validation, Cox regression, Research waste

## Abstract

**Background:**

Clinical prediction rules (CPRs) should be externally validated by independent researchers. Although there are many cardiovascular CPRs, most have not been externally validated. It is not known why some CPRs are externally validated by independent researchers and others are not.

**Methods:**

We analyzed cardiovascular risk CPRs included in a systematic review. Independent external validations were identified by forward citation searches of derivation studies. Time between the publication of a cardiovascular CPR and the first independent external validation was calculated. We assessed Kaplan-Meier estimates of the probability to have an independent external validation. Using univariable Cox regression, we explored whether characteristics of derivation (design, location, sample size, number of predictors, presentation format, validation in derivation), reporting (participants, predictors, outcomes, performance measure, information for risk calculation), and publication (journal impact factor) are associated with time to the first independent external validation.

**Results:**

Of 125 cardiovascular risk CPRs, 29 had an independent external validation. The median follow-up was 118 months (95% CI, 99–130). The 25th percentile of event time was 122 months (95% CI, 91–299). Cardiovascular risk CPRs from the USA were 4.15 times (95% CI, 1.89–9.13) more likely to have an independent external validation. Increasing the sample size of derivation by ten times was associated with a 2.32-fold (95% CI, 1.37–3.91) increase in the probability of having an independent external validation. CPRs presented with an internal validation tend to get an independent external validation sooner (HR = 1.73, 95% CI, 0.77–3.93). CPRs reporting all the information necessary for calculating individual risk were 2.65 (95% CI, 1.01–6.96) times more likely to have an independent external validation. Publishing a cardiovascular risk CPR in a journal that has one unit higher impact factor was associated with a 6% (95% CI, 3–9) higher likelihood of an independent external validation.

**Conclusions:**

The probability for cardiovascular risk CPRs to get an independent external validation was low even many years after their derivations. Authors of new cardiovascular risk CPRs should consider using adequate sample size, conducting an internal validation, and reporting all the information needed for risk calculation as these features were associated with an independent external validation.

**Electronic supplementary material:**

The online version of this article (10.1186/s41512-018-0025-6) contains supplementary material, which is available to authorized users.

## Background

It is important for clinicians to know that a clinical prediction rule (CPR) will accurately predict an outcome when applied to their patients. Although an internal validation using techniques such as cross-validation or bootstrapping may be included in derivation studies [1, 2], it only tests the reproducibility of a CPR and does not provide any information about whether the CPR will perform well in different populations [3, 4]. Therefore, the generalizability of a CPR should be confirmed in external validation studies by testing the performance of the CPR in new populations [3, 4].

At times, external validation studies are either published as a part of derivation studies or conducted later by researchers involved in developing the CPR. Systematic reviews have shown that CPRs tend to perform better in external validation studies done by researchers involved in developing them [5, 6]. The results of these external validation studies can be misleading because researchers may have intentionally and unintentionally led the CPRs they developed to perform more favorably [6, 7]. Ideally, a CPR’s performance should be evaluated in external validation studies conducted by researchers that have no conflict of interest with authors of the derivation study.

Many CPRs for various cardiovascular conditions have been developed, but most cardiovascular CPRs have not been externally validated [2, 8, 9]. CPRs that have been externally validated have often been done by researchers involved in deriving the CPR [2, 6]. Without reliable external validations, any use of a CPR in practice cannot be fully evidence-based. However, it is unknown how often and quickly cardiovascular CPRs are externally validated by independent researchers or why some cardiovascular CPRs are validated by independent researchers and others are not.

Therefore, we estimated the probability of having an independent external validation of a newly developed cardiovascular CPR and explored whether features of derivation, reporting, and publication of cardiovascular CPRs are associated with an independent external validation.

## Methods

### Source of data

We evaluated all cardiovascular risk CPRs included in a systematic review. The full description of the systematic review can be found elsewhere [2], but the search methods and selection criteria for derivation studies of cardiovascular risk CPRs are briefly summarized here. The authors of the systematic review searched Medline and Embase for articles that developed prognostic CPRs for cardiovascular disease published between 2004 and 2013. They also checked the reference lists of systematic reviews found in the electronic database search to look for articles that developed cardiovascular risk CPRs published before 2004. A study was eligible if it developed a multivariable model estimating risk of an arterial cardiovascular disease event in general population, developed a prediction model estimating risk of individual patients, and was written in English. They excluded a study if it only assessed the incremental value of adding new predictors to an existing CPR, developed a CPR for a venous cardiovascular disease event (e.g., Wells’ criteria for deep vein thrombosis), or developed a CPR for a specific population such as patients with diabetes, HIV, or atrial fibrillation.

For our study, we considered derivation studies that included multiple versions of a prediction model as one cardiovascular CPR because external validation studies often do not specify which version is evaluated. For example, Wilson et al. [10] published Framingham coronary heart disease risk equations and point scoring systems for men and women in a derivation study and they were treated as one coronary heart disease risk CPR, the Framingham Wilson coronary heart disease risk model.

### Outcome

We assessed the time interval measured in months between the publication of a derivation study and the first independent external validation. Independent external validation was defined as an external validation study conducted by investigators who have no conflict of interest with authors of the derivation study. We classified a study as an “independent external validation,” when (1) a CPR was applied to a new population different from the derivation, (2) a performance measure such as discrimination or calibration was reported, (3) no author overlapped with the authors of the derivation study, (4) no author had prior history of co-authorship with the authors of the derivation study, and (5) no other potential conflict of interest was identified after reviewing the author affiliation, funding source, acknowledgement, and conflict of interest statement. We excluded studies that applied a CPR to assess the risk of a different type of outcome (e.g., coronary heart disease risk score applied to assess the risk of atrial fibrillation), compared risks estimated by one CPR with another CPR, or used a modified version of a CPR.

In August of 2016, we conducted forward citation searches of all derivation studies of cardiovascular risk CPRs included in the systematic review using Scopus. For the Adult Treatment Panel III model, we used the executive summary of the Third Report of The National Cholesterol Education Program (NCEP) Expert Panel on Detection, Evaluation, And Treatment of High Blood Cholesterol In Adults [11] in addition to the final report [12] in the forward citation search because the executive summary was published 1 year earlier with a full description of the model. For each cardiovascular risk CPR, one of the authors (JW) screened titles and abstracts of retrieved references in chronological order and full text articles of potentially eligible references were reviewed. This process was continued until the first independent external validation study for the cardiovascular risk CPR was identified. Cardiovascular risk CPRs that had no independent external validation by the time of the forward citation search (August of 2016) were right censored.

The reference list of the systematic review by Damen et al. [2] was also reviewed to identify independent external validation studies. Using Pubmed, we verified publication dates of all derivation and validation studies. History of past co-authorship was investigated using the “Advanced” search option in Scopus.

### Predictors of an independent external validation

Because little is known about what predicts an independent external validation, we developed a list of predictors that might be associated with an independent external validation by considering how CPRs are developed, reported, and published.

Firstly, we reviewed features of CPR derivation that might be important to researchers planning an external validation [13] and selected the following six characteristics of derivation: study design, geographic location, sample size, number of predictors, presentation format, and validation in derivation. A cohort study is an ideal design when deriving a CPR. We determined a case-control design which was used when the development of an outcome was verified before the prediction was made [14]. We used the United Nation’s standard country or area codes for statistical use (M49) [15] to define geographical regions where CPRs were developed. Some derivation studies created more than one version of a CPR, and we used the predictors included in the full model to define the number of predictors. We determined a user-friendly format which was used when a CPR was presented with a simplified format for a risk calculation such as scoring system, chart, or online calculator. A derivation study may include internal or external validation. Internal validations assess a CPR’s reproducibility using techniques such as split sample, cross-validation, or bootstrapping, and external validations assess a CPR’s performance in a new population different from that of derivation study [4, 16–18]. An external validation may be included in a derivation study with or without an internal validation.

Secondly, we reviewed the Transparent Reporting of a multivariable model for Individual Prognosis or Diagnosis (TRIPOD) statement [19] to identify reporting items of derivation studies that might be essential for conducting an external validation study. We assessed whether authors clearly described participants (eligibility criteria, settings, and key characteristics), predictors (including how and when they were measured), outcomes (including how and when they were measured), performance measure (such as discrimination or calibration), and information for risk calculation (a constant and all regression coefficients or a scoring system with probabilities of an outcome needed for calculating individual risks was provided).

Lastly, we hypothesized that the impact factor of the journal in which CPRs are published might influence the chance of having an independent external validation. We used the impact factor reported in 2015 Thompson Reuters Journal Citation Index. A list of potential predictors and their definitions are presented in Additional file 1.

### Statistical analysis

We applied a logarithmic transformation to the sample size of derivation studies because it had a very skewed distribution. Only a small number of cardiovascular risk CPRs from continental Europe, the UK, Asia, and other geographic areas had an independent validation and these categories were combined. The derivation studies with missing information about predictor variables were excluded from each corresponding analysis.

The probability for a cardiovascular risk CPR to have an independent external validation was estimated using the Kaplan-Meier method. We reported the 25th percentile of event time because the cumulative probability of event (independent external validation) never reached 50%. The median time from publication of a cardiovascular risk CPR to date of our forward citation search (median follow-up time) was estimated according to the reverse Kaplan-Meier method [20, 21] to show whether the cardiovascular CPRs were followed up long enough after their derivations for the assessment of independent external validation.

We used Cox proportional hazards regression to evaluate the association between potential predictors and the time interval between a derivation of a cardiovascular CPR and the first independent external validation. Hazard ratios (HRs) and their 95% confidence intervals (CIs) were estimated in univariable Cox proportional hazards regression models. In addition, we graphically compared exposure group by plotting Kaplan-Meier estimates for the probability of an independent external validation for each level of categorical variables and each tertile of continuous variables. We focused on univariable analyses because the sample size prohibited evaluating predictor variables using a multivariable model. The proportional hazards assumption was tested using scaled Schoenfeld residuals [22], and no clear violation was detected. Stata (*Release 14*. College Station, TX: StataCorp LP) was used for all analyses.

## Results

**Fig. 1:**
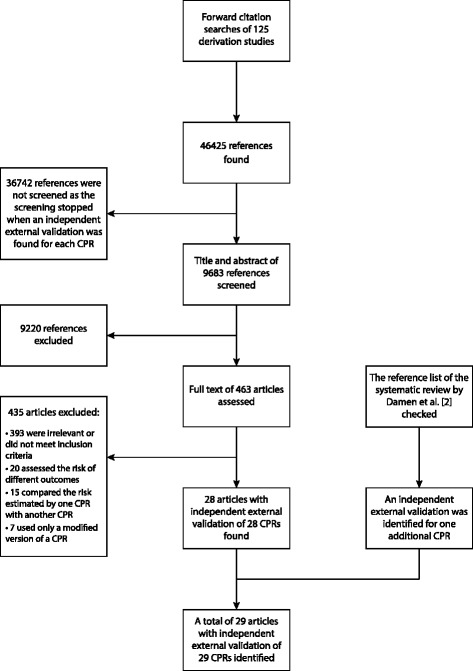
Flow diagram. Identifying cardiovascular risk clinical prediction rules (CPRs) with an independent external validation

Figure 1 summarizes how cardiovascular risk CPRs with an independent external validation study were identified. Of 125 cardiovascular risk CPRs we examined, 29 had independent external validation and 96 had no independent external validation (Fig. 1). For 33 cardiovascular CPRs, external validations that had no overlapping author with the derivation study were found. However, four of these CPRs only had external validation studies that included authors who had prior co-authorships with the authors of the derivation study.

The characteristics of cardiovascular risk CPRs included in our analyses are summarized in Table 1. Median derivation year of 125 cardiovascular risk CPRs was 2006 (IQR, 2002–2010). Median derivation year of cardiovascular risk CPRs that had an independent external validation was 2004 (IQR, 2002–2007), and those that had no independent external validation was 2007 (2003–2010). There was one derivation study [12] for which study design and sample size could not be determined. For another study [23], the number of predictors could not be determined.

**Table 1:** Characteristics of cardiovascular risk clinical prediction rules (CPRs)

	CPRs with an independent external validation, *n* = 29	CPRs with no independent external validation, *n* = 96	All cardiovascular risk CPRs, *n* = 125
A. CPR derivation			
1. Study design, *n* (%)*			
Cohort	25 (89.3)	85 (88.5)	110 (88.7)
Case-control	3 (10.7)	11 (11.5)	14 (11.3)
2. Geographic location, *n* (%)			
USA	20 (69.0)	23 (24.0)	43 (34.4)
Continental Europe	4 (13.8)	25 (26.0)	29 (23.2)
UK	2 (6.9)	10 (10.4)	12 (9.6)
Asia	0 (0.0)	19 (19.8)	19 (15.2)
Other	0 (0.0)	10 (10.4)	10 (8.0)
Multiple countries	3 (10.3)	9 (9.4)	12 (9.6)
3. Sample size, median (IQR)*	6032 (5277–19,306)	5206 (2721–12,299.5)	5722.5 (3396–12,711.5)
4. Number of predictors, median (IQR)**	8 (7–9)	8 (6–9)	8 (6–9)
5. Presentation format, *n* (%)			
User-friendly	15 (51.7)	37 (38.5)	52 (41.6)
Not user-friendly	14 (48.3)	59 (61.5)	73 (58.4)
6. Validation in derivation, *n* (%)			
External	1 (3.5)	18 (18.8)	19 (15.2)
Internal	9 (31.0)	23 (24.0)	32 (25.6)
None	19 (65.5)	55 (57.3)	74 (59.2)
B. Reporting and publication			
1. Description of participants, *n* (%)			
Clear	15 (51.7)	46 (47.9)	61 (48.8)
Unclear	14 (42.3)	50 (52.1)	64 (51.2)
2. Description of predictors, *n* (%)			
Clear	11 (37.9)	54 (56.3)	65 (52.0)
Unclear	18 (62.1)	42 (43.8)	60 (48.0)
3. Description of outcomes, *n* (%)			
Clear	8 (27.6)	40 (41.7)	48 (38.4)
Unclear	21 (72.4)	56 (58.3)	77 (61.6)
4. Performance measure, *n* (%)			
Reported	16 (55.2)	67 (69.8)	83 (66.4)
Not reported	13 (44.8)	29 (30.2)	42 (33.6)
5. Information for risk calculation, *n* (%)			
Reported	24 (82.8)	54 (56.3)	78 (62.4)
Not reported	5 (17.2)	42 (43.8)	47 (37.6)
6. Impact factor, median (IQR)	15.1 (4.3–17.2)	3.9 (3.1–7.1)	4.3 (3.2–15.1)

*Could not be determined for Adult Treatment Panel III model (reference)

**Could not be determined for CHD prevention model by McNeil et al. (reference)

Derivation studies were most frequently published in Circulation (*n* = 14) followed by the BMJ (*n* = 7). The American Heart Journal, the American Journal of Cardiology, and the European Journal of Cardiovascular Prevention and Rehabilitation each published five derivation studies. Independent external validation studies were most frequently published in the BMJ (*n* = 3) and the American Journal of Cardiology (*n* = 3). The median impact factor of journals that published the independent external validation studies of cardiovascular risk CPRs was 5.1 (IQR, 3.4–8.9). The full list of journals that published derivation studies of cardiovascular risk CPRs and their independent external validation studies is provided in Additional file 2.

**Fig. 2:**
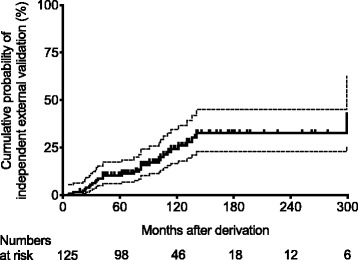
Probability for a cardiovascular risk clinical prediction rule (CPR) to have an independent external validation

Kaplan-Meier estimates of the probability for a cardiovascular risk CPR to have an independent external validation are illustrated in Fig. 2. The median time from publication of a cardiovascular risk CPR to date of our forward citation search (median follow-up time) was 118 months (95% CI, 99–130). We found that it took 122 months (95% CI, 91–299) before the probability of a CPR to have an independent external validation reached 25%. A coronary heart disease risk score by Polonsky et al. [24] had the shortest interval of 6 months until the first independent external validation. All independent external validations were done before 142 months except for a coronary heart disease risk score by Wilson et al. [25] which took 299 months until the first independent external validation. The cumulative probability of having an independent external validation at 60, 120, and 180 months after derivation of a cardiovascular risk CPR was 10.5% (95% CI, 6.2–17.4), 24.3% (95% CI, 16.7–34.6), and 32.6% (95% CI, 22.9–45.1), respectively.

### Univariable analysis

**Fig. 3:**
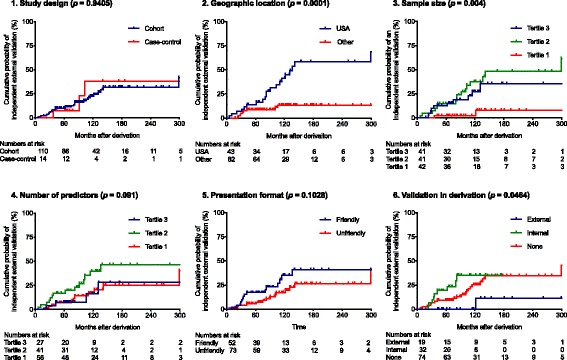
Kaplan-Meier plot. Probability of an independent external validation for derivation-related predictor variables

**Fig. 4:**
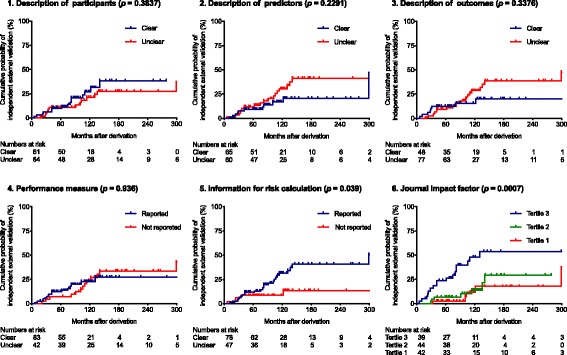
Kaplan-Meier plot. Probability of an independent external validation for reporting and publication-related predictor variables

The results of univariable Cox proportional hazards regression analyses are presented in Figs. 3 and 4 and Table 2. Three of six features of CPR derivation studies assessed were associated with having an independent external validation: geographic location (HR for USA = 4.15, 95% CI 1.89–9.13), sample size (HR = 2.32, 95% CI 1.37–3.91), and validation in derivation (HR for internal validation = 1.73, 95% CI 0.77–3.90). A post hoc sensitivity analysis showed that the HR for cardiovascular risk CPRs derived in the USA (United States of America) excluding 26 cardiovascular risk CPRs developed by Framingham Heart Study researchers was 2.46 (95% CI, 0.92–6.61, *p* = 0.0842) compared to cardiovascular risk CPRs derived elsewhere.

**Table 2:** Univariable Cox proportional hazards regression analyses

	Hazard ratio	(95% CI)	*p* value
A. CPR derivation			
1. Study design			0.9409
Cohort	0.96	(0.29–3.17)	
Case-control	1	–	
2. Geographic location			0.0002
USA	4.15	(1.89–9.13)	
Other	1	–	
3. Log_10_(sample size)	2.32	(1.37–3.91)	0.0035
4. Number of predictors	1.01	(0.92–1.12)	0.7833
5. Presentation format			0.1095
User-friendly	1.82	(0.88–3.79)	
Not user-friendly	1	–	
6. Validation in derivation			0.0257
External	0.19	(0.03–1.42)	
Internal	1.73	(0.77–3.90)	
None	1	–	
B. Reporting and publication			
1. Description of participants			0.3853
Clear	1.39	(0.66–2.94)	
Unclear	1	–	
2. Description of predictors			0.2269
Clear	0.63	(0.30–1.34)	
Unclear	1		
3. Description of outcomes			0.3282
Clear	0.67	(0.30–1.52)	
Unclear	1		
4. Performance measure			0.9360
Reported	1.03	(0.48–2.20)	
Not reported	1		
5. Information for risk calculation			0.0289
Reported	2.65	(1.01–6.96)	
Not reported	1		
6. Impact factor	1.06	(1.03–1.09)	0.0002

Of six reporting and publication-related features analyzed, reporting information for risk calculation (HR = 2.65, 95% CI 1.01–6.96) and publishing the derivation study in a journal with higher impact factor (HR = 1.06, 95% CI 1.03–1.09) were associated with having an independent external validation.

## Discussion

### Summary of results

In this study, we examined the probability of having an independent external validation of a newly developed cardiovascular CPR and explored whether 12 characteristics of derivation, reporting, and publication of cardiovascular risk CPRs are associated with independent external validation. We found most cardiovascular CPRs are not independently validated even 10 years after publication. This greatly limits the value of studies deriving new CPRs, because without strong evidence of validity, CPRs cannot make an evidence-based contribution to clinical practice. We found that CPRs derived in the USA were four times more likely to be externally validated by independent researchers although this is heavily influenced by multiple CPRs from the Framingham study. Besides geographic location, larger sample size and publishing in journals with higher impact factor are associated with shorter time to independent validation, as are providing information for risk calculation and internal validation results. These latter two at least are within the control of the derivation study authors and may provide a route for authors to increase the likelihood that their published CPRs will progress further along the pathway to evidence-based practice.

### Comparison with existing literature

Our findings are consistent with existing systematic reviews that most CPRs do not get externally validated by independent researchers [2, 5, 6]. However, this is the first study to assess the probability of having an independent external validation after CPRs are derived using survival analysis by taking censoring and time to event information into account. This is the first study to explore the factors that might influence the chance of having an independent external validation. We also applied a much stricter definition of independent external validation: no traceable conflict of interest with derivation authors.

We analyzed cardiovascular risk CPRs included in a systematic review by Damen et al. [2] which reported that 19% of cardiovascular risk CPRs had an independent external validation. Although we applied a stricter definition of independent external validation, we found that 23.2% of cardiovascular risk CPRs had an independent external validation. This is probably because we conducted forward citation searches of all cardiovascular risk CPRs included which allowed us to identify independent external validation studies more thoroughly than the search strategy of the systematic review.

Many systematic reviews have pointed out that quality of reporting in CPR research is poor [1, 6, 26–29]. Published in 2015, the Transparent Reporting of a multivariable model for Individual Prognosis or Diagnosis (TRIPOD) statement [19] provides a much needed guidance to authors. We chose five reporting features from the TRIPOD statement that might be particularly important to researchers externally validating cardiovascular risk CPRs and assessed whether they are associated with time to the first independent external validation. Although our study found such association in only one of five reporting features assessed, we strongly believe that clear reporting is crucial in reducing avoidable waste in many steps of CPR development.

### Strengths and limitations

We were able to ascertain complete data about predictor variables for almost all derivation studies: of 125 derivation studies, one had two missing variables and another had one missing variable. We also rigorously ascertained the outcome (presence of an independent external validation study) by conducting forward citation searches of all derivation studies.

We defined independent external validation as an external validation study conducted by investigators who have no conflict of interest with authors of the derivation study. We applied a stricter definition of independent external validation than previously used [2, 6] and attempted to identify all pragmatically searchable conflict of interest. However, some form of collaboration between authors of derivation and external validation may not have been traceable.

Some predictor variables under study were correlated: for example, the studies published in higher impact journals generally had the larger sample sizes. Further, the observations in the data set may not be fully independent, since a number of derivation studies originated from the same research group (e.g., Framingham Heart Study). The number of available cardiovascular risk CPRs and independent external validations in our data precluded assessing the predictors in a multivariable analysis that could account for these correlations. Therefore, any positive findings in our exploratory analyses should be interpreted cautiously, as hypothesis-generating, until they can be confirmed in multivariable analyses of a future, larger data set.

### Research implications

Although the number of studies reporting CPR research has been rapidly increasing [8, 9, 30], too much focus is still on creating new CPRs rather than externally validating and assessing the impacts of existing CPRs [6, 8, 13]. Cardiovascular risk CPR research has not been an exception [2]. Our study furthered the understanding of this problem by showing that the probability for cardiovascular risk CPRs to get externally validated by independent researchers is low even many years after they are created. Clinicians do not know how well most cardiovascular risk CPRs perform in new populations, and these cardiovascular risk CPRs are unlikely to be used in practice.

Researchers interested in developing a new cardiovascular risk CPR should systematically review existing evidence and assess whether a new CPR is needed [13]. When creating a new cardiovascular CPR is clearly justified, it should be created using proper design and rigorous methods to avoid adding redundant CPRs. Based on the TRIPOD statement, all important information should be unambiguously described so that others can validate, update, implement, and use the CPR. Particularly, authors should consider using adequate sample size, conducting an internal validation, and reporting all the information needed for individual risk calculation as these might improve the probability of having an independent external validation.

Independent external validation studies of cardiovascular risk CPRs seemed to be published in journals with lower median impact factor (5.1, IQR, 3.4–8.9) than their derivation studies (15.1, IQR, 4.3–17.2). Well-conducted independent external validation studies deserve closer attention by journal editors especially in the presence of many existing CPRs.

## Conclusion

The cumulative probability of having an external validation by independent researchers was low even many years after the derivation of cardiovascular risk CPRs. Authors of new cardiovascular risk CPRs should use adequate sample size, conduct an internal validation, and unambiguously report all the information needed for risk calculation as these features were associated with an independent external validation. Publishing cardiovascular risk CPRs in journals with high impact factor may also improve the chance of an independent external validation.

## Additional files

Additional file 1: Potential predictors for independent external validation. (DOCX 113 kb)

Additional file 2: Journals that published derivation and independent external validation studies. (DOCX 121 kb)

## Abbreviations

CI: Confidence interval
CPR: Clinical prediction rule
HR: Hazard ratio
IQR: Interquartile range
UK: United Kingdom
US: United States
