# Supplementary material for: Predictors for independent external validation of cardiovascular risk clinical prediction rules: Cox proportional hazards regression analyses
Source: Diagn Progn Res. 2018 Feb 6;2:3. doi: 10.1186/s41512-018-0025-6 (PMC6460844; doi:10.1186/s41512-018-0025-6)
Supplement: Supplementary file 1 — Potential predictors for independent external validation. (DOCX 113 kb) [file 41512_2018_25_MOESM1_ESM.docx]

**Table S1.** Potential predictors for cardiovascular risk clinical prediction rules (CPRs) to have an independent external validation.

| **Predictor variable** | **Definition** |
| --- | --- |
| **A. CPR derivation** |  |
| 1. Study design |  |
| Cohort | A prediction was made before an outcome is verified. |
| Case-control | An outcome was verified before a prediction is made. |
| 2. Geographic location | The country or region where a CPR was developed. |
| 3. Sample size | The number of participants used to develop a CPR. |
| 4. Number of predictors | The number of predictors included in the largest model. |
| 5. Presentation format |  |
| Friendly | A simplified format for a risk calculation (e.g. scoring system, chart, online calculator) was provided. |
| Unfriendly | A simplified format for a risk calculation was not provided. |
| 6. Validation in derivation |  |
| External | CPR’s performance was assessed in a new population different from that of derivation study. |
| Internal | CPR’s performance was assessed using split sample, cross-validation, or bootstrapping. |
| None | No internal or external validation was conducted. |
|  |  |
| **B. Reporting and publication** |  |
| 1. Description of participants |  |
| Clear | Eligibility criteria, settings and key characteristics of participants were clearly described. |
| Unclear | Participants were unclearly described. |
| 2. Description of predictors |  |
| Clear | All predictors were clearly described including how and when they were measured. |
| Unclear | Predictors were unclearly described. |
| 3. Description of outcomes |  |
| Clear | Outcomes were clearly described including how and when they were assessed. |
| Unclear | Outcomes were unclearly described. |
| 4. Performance measure |  |
| Reported | A CPR’s performance measure such as discrimination or calibration was reported. |
| Not reported | No performance measure was reported. |
| 5. Information for risk calculation |  |
| Reported | All the information (e.g. a constant and all regression coefficients, a scoring system with probabilities of an outcome) needed for calculating individual risks was provided. |
| Not reported | Information provided was insufficient for risk calculation. |
| 6. Journal impact factor | The Impact factor reported in 2015 Thompson Reuters Journal Citation Index. |
