# Supplementary material for: Predictors for independent external validation of cardiovascular risk clinical prediction rules: Cox proportional hazards regression analyses
Source: Diagn Progn Res. 2018 Feb 6;2:3. doi: 10.1186/s41512-018-0025-6 (PMC6460844; doi:10.1186/s41512-018-0025-6)
Supplement: Supplementary file 2 — Journals that published derivation and independent external validation studies. (DOCX 121 kb) [file 41512_2018_25_MOESM2_ESM.docx]

**Table S2.** Number of derivation and independent external validation studies of cardiovascular risk prediction rules published by journals.

| **Derivation study** | **Journal** |
| --- | --- |
| 14 | Circulation |
| 7 | The BMJ |
| 5 | The American Heart Journal, the American Journal of Cardiology, the European Journal of Cardiovascular Prevention and Rehabilitation |
| 4 | The Journal of Clinical Epidemiology, Preventive Medicine, Stroke |
| 3 | Atherosclerosis, Circulation Journal, the European Heart Journal, the European Journal of Epidemiology, Heart, the International Journal of Epidemiology, the JAMA, the Journal of Cardiovascular Risk, the Journal of Epidemiology and Community Health |
| 2 | Annals of Internal Medicine, Diabetes Care, the European Journal of Preventive Cardiology, Hypertension Research, Italian Heart Journal, the Journal of the American College of Cardiology, Journal of Chronic Diseases, the Journal of Epidemiology, the Lancet, Nutrition, Metabolism & Cardiovascular Diseases, PLOS ONE |
| 1 | Acta Cardiologica, the American Journal of Hypertension, the American Journal of Preventive Medicine, Australian and New Zealand Journal of Public Health, BMC Research Notes, Cardiovascular Diabetology, Cerebrovascular Diseases, Clinical Chemistry, Clinical Endocrinology, Current Medical Research & Opinion, Diabetologia, the European Journal of Clinical Investigation, Heart International, the Hellenic Journal of Cardiology, Hypertension, the International Journal of Cardiology, the International Journal of Obesity, International Journal of Stroke, the Journal of the American Society of Hypertension, Journal of Atherosclerosis and Thrombosis, the Journal of Electrocardiology, Journal of Hypertension, the Journal of Nutrition, the Laryngoscope, the Medical Journal of Australia, Neurological Sciences, Public Health Nursing, Statistics in Medicine |
| **Independent external validation study** | **Journal** |
| 3 | The American Journal of Cardiology, the BMJ |
| 2 | Diabetes Care, the European Journal of Cardiovascular Prevention and Rehabilitation, the Journal of the American College of Cardiology, the Journal of Epidemiology and Community Health, Stroke |
| 1 | The American Journal of Medicine, Annals of Internal Medicine, Atención Primaria, Circulation, Heart, Hypertension, Hypertension Research, Indian Journal of Community Health, the International Journal of Cardiology, Journal of hypertension, the Medical journal of Australia, Neuroepidemiology, Nutrition, Metabolism & Cardiovascular Diseases |
